# Establishing induced pluripotent stem cell lines from two dominant optic atrophy patients with distinct *OPA1* mutations and clinical pathologies

**DOI:** 10.3389/fgene.2023.1251216

**Published:** 2023-09-04

**Authors:** Katherine A. Pohl, Xiangmei Zhang, Anh H. Pham, Jane W. Chan, Alfredo A. Sadun, Xian-Jie Yang

**Affiliations:** ^1^ Department of Ophthalmology, Stein Eye Institute, University of California, Los Angeles, Los Angeles, CA, United States; ^2^ Molecular Biology Institute, University of California, Los Angeles, Los Angeles, CA, United States; ^3^ Department of Ophthalmology, Doheny Eye Institute, University of California, Los Angeles, Pasadena, CA, United States

**Keywords:** dominant optic atrophy, DOA, OPA1, induced pluripotent stem cells (iPSC), retinal ganglion cell, RGC

## Abstract

Dominant optic atrophy (DOA) is an inherited disease that leads to the loss of retinal ganglion cells (RGCs), the projection neurons that relay visual information from the retina to the brain through the optic nerve. The majority of DOA cases can be attributed to mutations in *optic atrophy 1* (*OPA1*), a nuclear gene encoding a mitochondrial-targeted protein that plays important roles in maintaining mitochondrial structure, dynamics, and bioenergetics. Although *OPA1* is ubiquitously expressed in all human tissues, RGCs appear to be the primary cell type affected by *OPA1* mutations. DOA has not been extensively studied in human RGCs due to the general unavailability of retinal tissues. However, recent advances in stem cell biology have made it possible to produce human RGCs from pluripotent stem cells (PSCs). To aid in establishing DOA disease models based on human PSC-derived RGCs, we have generated iPSC lines from two DOA patients who carry distinct *OPA1* mutations and present very different disease symptoms. Studies using these *OPA1* mutant RGCs can be correlated with clinical features in the patients to provide insights into DOA disease mechanisms.

## Introduction

Dominant optic atrophy (DOA; OMIM #165500) is the most prevalent inherited optic neuropathy, affecting an estimated 1:10,000 to 1:50,000 individuals worldwide (Kivlin et al., 1983; Kjer et al., 1996; Yu-Wai-Man et al., 2010a; Yu-Wai-Man et al., 2011). DOA is characterized by the preferential degeneration of retinal ganglion cells (RGCs), whose axons make up the optic nerve. RGC loss causes progressive bilateral vision loss that often begins in the first decade of life. A hallmark of DOA which aids in its diagnosis is wedge-shaped, temporal optic nerve head pallor (Amati-Bonneau et al., 2009). Other clinical symptoms include reduced visual acuity, color vision deficits, and central visual field defects (Kjer, 1959; Eliott et al., 1993; Cohn et al., 2007; Yu-Wai-Man et al., 2010a; Han et al., 2022). DOA patients also tend to have smaller optic nerves, suggesting that they may be born with fewer RGCs (Barboni et al., 2010; Barboni et al., 2011).

The majority of DOA cases, ∼60–80%, are caused by mutations in the gene optic atrophy 1 (*OPA1*; OMIM: *605290), a nuclear gene that encodes a dynamin-related GTPase located within the mitochondria (Cohn et al., 2007; Alexander et al., 2000; Delettre et al., 2000; Ferré et al., 2009). The OPA1 protein plays important roles in mitochondrial fusion, cristae remodeling, and bioenergetic output (Olichon et al., 2003; Cipolat et al., 2004; Griparic et al., 2004; Frezza et al., 2006; Olichon et al., 2007; Zanna et al., 2008; Agier et al., 2012; Cogliati et al., 2013; Ramonet et al., 2013; Lee et al., 2017; Quintana-Cabrera et al., 2018). To date, over 500 different pathogenic mutations throughout the ∼100 kb *OPA1* gene have been documented (http://www.LOVD.nl/OPA1) (Le Roux et al., 2019). Although autosomal dominant, *OPA1* mutations are ∼43–88% penetrant (Toomes et al., 2001; Cohn et al., 2007). Patients range in their disease presentation, even within families harboring the same mutation, from asymptomatic to legally blind (Hoyt, 1980; Delettre et al., 2002; Yu-Wai-Man et al., 2010a). A subset of patients develops extraocular features including sensorineural hearing loss, ataxia, myopathy, and peripheral neuropathy. These patients are classified as having DOA+ (OMIM #125250, 165500), a diagnosis that has been correlated with missense mutations in the GTPase domain of OPA1 (Hoyt, 1980; Treft et al., 1984; Amati-Bonneau et al., 2008; Yu-Wai-Man et al., 2010b; Maeda-Katahira et al., 2019). In extremely rare cases, individuals have been found to harbor biallelic (usually compound heterozygous) *OPA1* mutations (Carelli et al., 2015). These individuals are usually classified as having Behr syndrome (OMIM #210000) a severe neurological disease with symptoms that include advanced early-onset optic atrophy, ataxia, peripheral neuropathy, pyramidal signs, dysarthria, intellectual disability, and metabolic stroke (Schaaf et al., 2011; Bonneau et al., 2014; Lee et al., 2016; Nasca et al., 2017; Zerem et al., 2019; Othman et al., 2022).

Interestingly, although *OPA1* is expressed ubiquitously, most DOA patients only exhibit signs and symptoms related to RGC loss causing optic neuropathy. Due to the limited availability of human retinal tissues, non-human cells or human cell types other than RGCs have been used to study *OPA1* in relation to DOA (Lodi et al., 2004; Alavi et al., 2007; Olichon et al., 2007; Song et al., 2007; Zanna et al., 2008; Alavi et al., 2009; White et al., 2009; Dayanithi et al., 2010; Heiduschka et al., 2010; Williams et al., 2010; van Bergen et al., 2011; Williams et al., 2012; Rahn et al., 2013; Varanita et al., 2015; Chao de la Barca et al., 2017; Chao de la Barca et al., 2020). Recent advances in stem cell technology have enabled the derivation of human retinal neurons from pluripotent stem cells (PSCs) (Osakada et al., 2009; Ohlemacher et al., 2015; Sluch et al., 2017; Rabesandratana et al., 2020; Zhang X. et al., 2021), permitting *in vitro* DOA disease models to be established using patients’ induced pluripotent stem cells (iPSCs). However, the heterogeneity of *OPA1* genotypes and phenotypes present in the DOA patient population, coupled with the incomplete penetrance of the disease, necessitates that a range of PSC lines containing distinct *OPA1* mutations be generated to properly model and understand *OPA1*-driven DOA. To help establishing DOA models that recapitulate the spectrum of the disease, we generated iPSC lines from two DOA patients with heterozygous *OPA1* mutations that are distinct from those in previously reported iPSC lines (Chen et al., 2016; Galera-Monge et al., 2016; Zurita-Diaz et al., 2017; Jonikas et al., 2018; Zhang XH. et al., 2021; Sladen et al., 2021; Chan et al., 2022; Sladen et al., 2022; Sun et al., 2022). Importantly, we also describe the clinical presentation of both DOA patients at the time of iPSC derivation, which is important information to consider when characterizing and validating disease models derived from these iPSC lines.

## Results

### Genetic and clinical presentation of DOA patients

Two DOA patients with previously determined, heterozygous *OPA1* mutations were recruited for the study. Both patients are male and of European ancestry. The first patient, referred to as “Patient 1,” presented with symptoms of DOA at age 7 and has a single G insertion in one allele of the *OPA1* gene exon 19, which is a part of the central dynamin region of the OPA1 protein. This insertional mutation causes a frameshift that leads to an early stop codon, resulting in a predicted protein product of 652 amino acids as opposed to the 1,015 amino acids encoded by the full-length wild type (WT) transcript (Table 1). The second patient, “Patient 2,” was relatively asymptomatic and found to have an *OPA1* mutation when his son with vision loss was diagnosed with DOA. Patient 2 has a heterozygous, two base pair AT deletion in exon 13, which encodes part of the GTPase domain of the OPA1 protein. This mutation also causes a frameshift and subsequent premature stop codon. The mutant transcript is predicted to encode a protein of 483 amino acids as opposed to the 1,015 amino acids encoded by the full-length WT transcript (Table 1).

**TABLE 1:** Summary of patients’ *OPA1* mutations and clinical symptoms.

*OPA1* genotype		
	Patient 1	Patient 2
Mutation	NM_130837.3:c.1948dup, NP_570850.2:p.(Glu650Glyfs*4)	NM_130837.3:c.1417_1418del, NP_570850.2:p.(Ile473Phefs*12)
Effect	Truncated protein (652 vs. 1,015 amino acids)	Truncated protein (483 vs. 1,015 amino acids)
Exon affected	19	13
Location	Central dynamin region	GTPase domain
Clinical Presentation		
	Patient 1	Patient 2
Visual Acuity	Right (OD): 20/100 -1 Left (OS): 20/150	Right (OD): 20/25 Left (OS): 20/25
IOP	OD 16, OS 15	OD 14, OS 14
Other	Slight hearing problems; cataract	Largely asymptomatic

At the time of enrollment and iPSC derivation, Patient 1 was 49 years old and Patient 2 was 60 years old. Neither patient was reported to have extraocular neurological disease or additional neural retina disorders. Additionally, both patients tested negative for bloodborne infectious diseases including hepatitis B, hepatitis C, HIV, and syphilis. The donor patients underwent a comprehensive ophthalmological examination in which visual acuity, tonometry, fundus imaging, Humphrey visual field (HVF) testing, and spectral-domain optical coherence tomography (SD-OCT) were performed.

**FIGURE 1:**
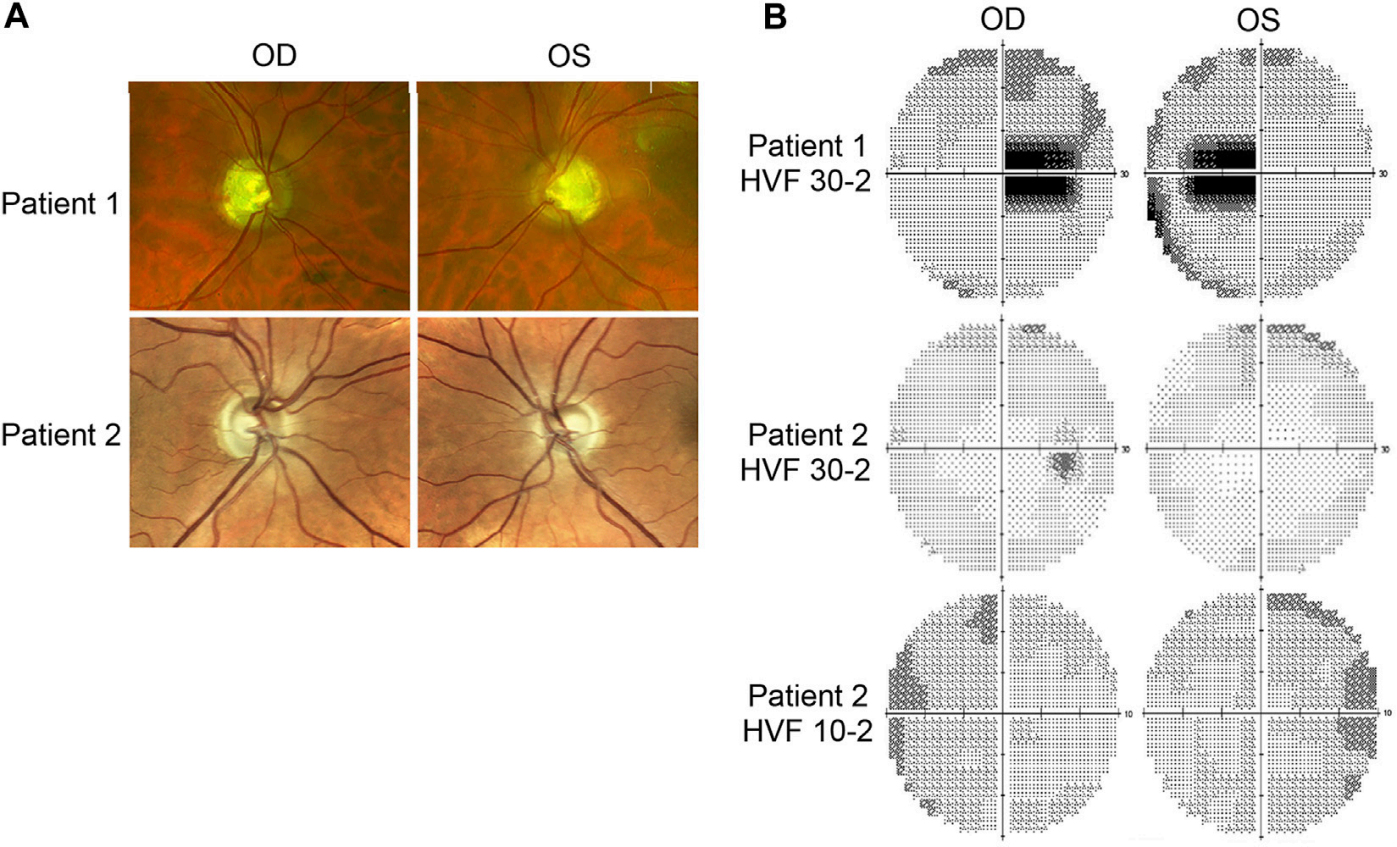
Fundus imaging and visual field testing. **(A)** Fundus images of Patient 1 and Patient 2 displaying bilateral, temporal optic nerve head pallor. **(B)** Humphrey visual field (HVF) testing reports. Patient 1’s HVF 30-2 showed bilateral, temporal, paracentral scotomas. Patient 2’s HVFs detected small paracentral scotomas. OD: *oculus dexter*, right eye; OS: *oculus sinister*, left eye.

**FIGURE 2:**
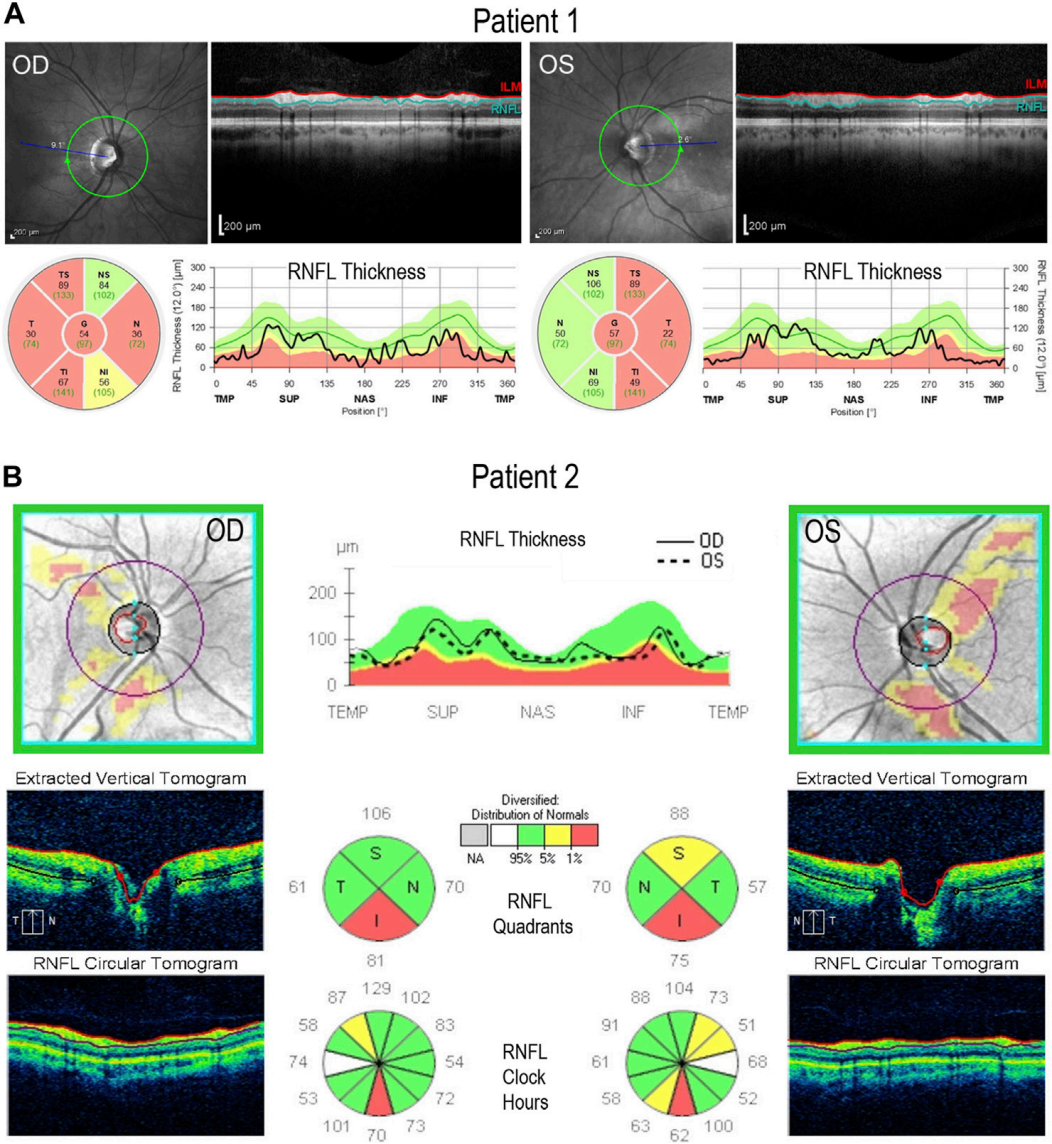
Retinal lamination imaging by optical coherence tomography. **(A)** Patient 1’s OCT results demonstrate bilateral retinal nerve fiber layer (RNFL) thinning, most significantly in the temporal zone, involving both superior and inferior regions. **(B)** Patient 2’s OCT results reveal bilateral, inferior RNFL thinning, accompanied by some superior fiber loss in both retinas. ILM, inner limiting membrane; RNFL, retinal nerve fiber layer; TMP (T); temporal; SUP (S): superior; INF (I): inferior; NAS (N): nasal.

Patient 1 displayed ophthalmologic symptoms of classical DOA. His visual acuity had declined to 20/100 -1 (OD) and 20/150 (OS) (Table 1), and fundus imaging showed typical, wedge-shaped retinal nerve fiber layer (RNFL) loss with temporal pallor (Figure 1A). HVF 30-2 testing revealed bilateral, cecocentral scotomas (Figure 1B). In addition, SD-OCT showed RNFL thinning temporal to the optic disc, with some additional thinning in the inferior and nasal portions of the optic disc in the right eye (Figure 2A).

In contrast, Patient 2 has remained largely asymptomatic over his lifetime. Despite fundus images that showed temporal pallor of the optic nerve, visual acuity was 20/25 in both eyes (Figure 1A; Table 1). Visual field assessment detected a small paracentral scotoma (Figure 1B), which made it difficult for Patient 2 to focus on the central target during Humphrey Visual Field (HVF 30-2) testing, resulting in high false positive and negative rates. The smaller field of HVF (10-2) testing allowed the patient to maintain central fixation on the target and detected the edge of the central scotomas, which are located superior temporally on HVF 10-2 (Figure 1B). SD-OCT revealed bilateral inferior RNFL thinning with more advanced superior and temporal thinning of the left eye (Figure 2B).

### Generation of *OPA1* heterozygous-mutant iPSCs from DOA patients

To generate iPSC lines from both DOA patients, peripheral blood mononuclear cells (PBMCs) were isolated from patients’ blood samples and reprogrammed by episomally expressing the pluripotency factors OCT3/4, SOX2, KLF4, L-Myc, shp53, Lin28, and SV40LT (Okita et al., 2013; Toombs et al., 2020). Two independent iPSC lines (n1a and n1b) were established from each patient. Lines generated from Patient 1 and Patient 2 were named 1iDOAn1a/1iDOAn1b, and 2iDOAn1a/2iDOAn1b, respectively. Although all four iPSC lines were authenticated, data in this report depicts the n1a lines only.

**FIGURE 3:**
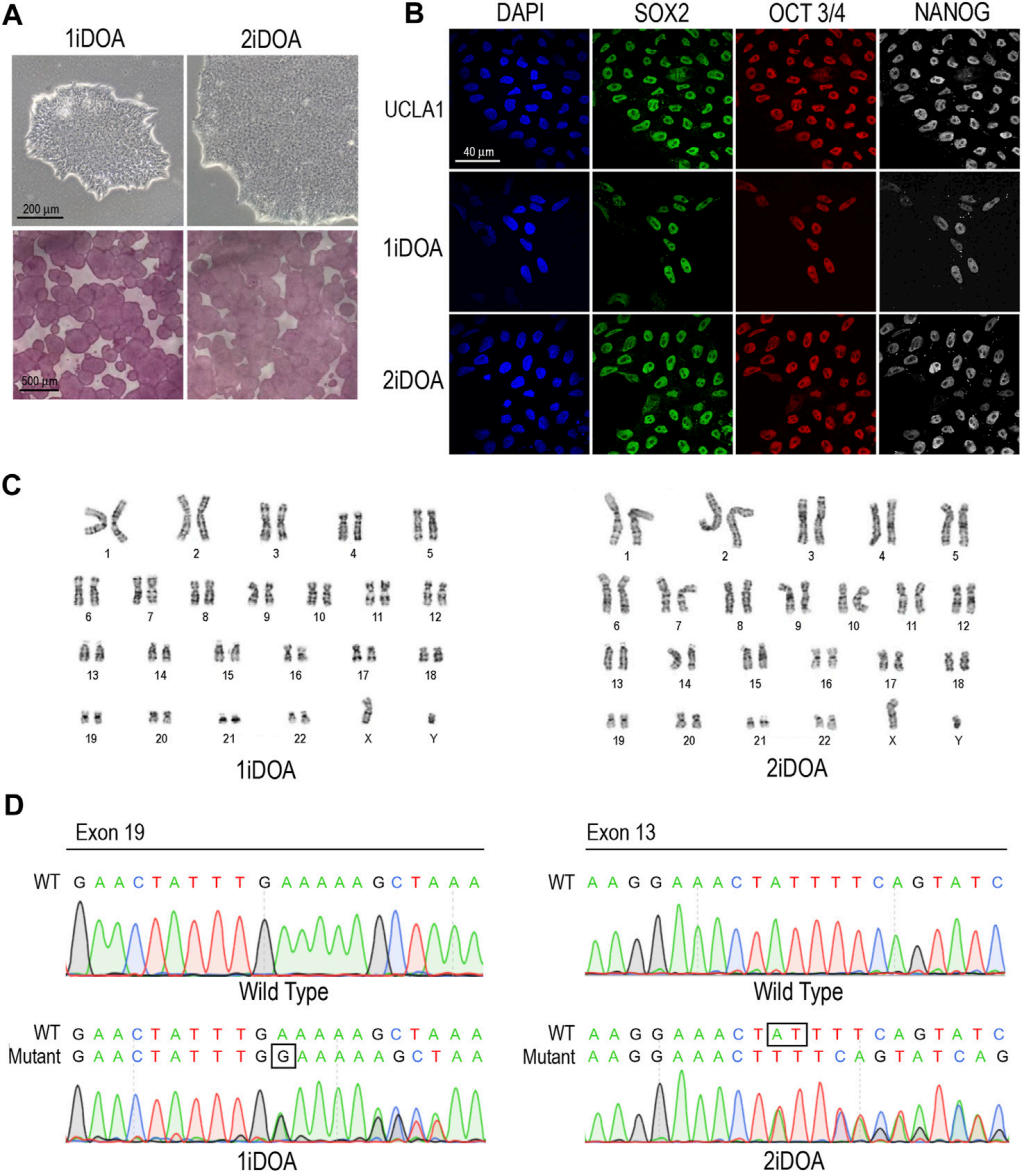
Characterization of *OPA1*-mutant iPSCs from DOA patients. **(A)** Brightfield images of iPSCs reprogrammed from DOA Patient 1 (1iDOA) and Patient 2 (2iDOA) display normal pluripotent stem cell morphology (top row) and stain positive for alkaline phosphatase (bottom row). Scale bars: 200 μm (top row), 500 μm (bottom row). **(B)** 1iDOA and 2iDOA iPSCs express the pluripotent stem cell markers SOX2, OCT3/4, and NANOG similarly to the wild type ESC line, UCLA1. Scale bar: 40 μm. **(C)** Both iPSC lines display a 46, XY normal male karyotype. **(D)** DNA sequences of iPSC lines 1iDOA and 2iDOA (bottom row) aligned to the corresponding regions of *OPA1* in the wild type (WT) reference genome (top row). Sequences confirm that 1iDOA contains a heterozygous, single base pair (G) insertion in exon 19 and that 2iDOA has a heterozygous, 2 base pair (AT) deletion in exon 13. Peaks reflect both WT and Mutant allele bases at a given position. Allele sequence identities are clarified above peaks. Boxed regions indicate bases inserted (1iDOA, mutant allele) or deleted (2iDOA, WT allele).

Both 1iDOA and 2iDOA displayed typical pluripotent stem cell morphology and stained positive for alkaline phosphatase (AP) activity (Figure 3A). Immunocytochemistry was performed to further verify the expression of pluripotency markers by the iPSCs in comparison to an established human embryonic stem cell (ESC) line, UCLA1. All lines were shown to similarly co-express the PSC markers SOX2, OCT3/4, and NANOG (Figure 3B). Furthermore, 1iDOA and 2iDOA iPSCs displayed a normal male karyotype with 22-pair autosomes and XY chromosomes (Figure 3C). The derived iPSC lines tested negative for *mycoplasma* and were characterized by the short tandem repeat (STR) analysis to establish their genetic identities (not shown). To confirm that the iPSC lines carried the DOA patients’ specific *OPA1* mutations, genomic DNA was extracted from 1iDOA and 2iDOA iPSCs and used as templates to obtain PCR products spanning the expected mutation sites in exons 19 and 13, respectively. DNA sequencing analysis of the PCR products validated that each patient’s existing heterozygous *OPA1* mutation was present in the given iPSC lines (Figure 3D).

### Detection of ROS production in PSC lines

**FIGURE 4:**
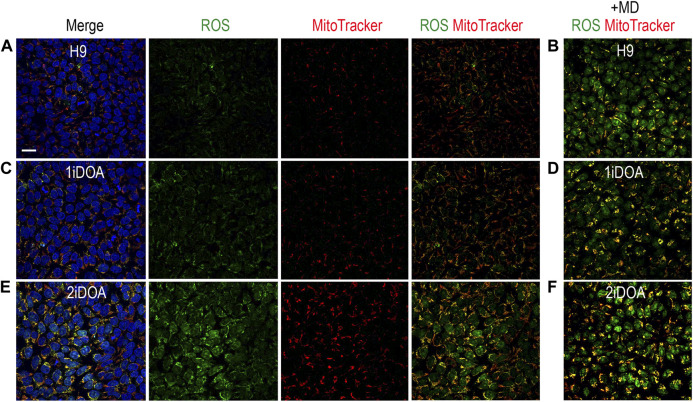
Detection of ROS production in PSC lines. Detection of mitochondria and cellular ROS in pluripotent stem cell (PSC) lines were performed using live cells. **(A, C, E)** show representative signals of ROS (green), MitoTracker (red) and merged images for 1iDOA, 2iDOA, and the control ESC H9 lines. **(B, D, F)** show merged ROS and MitoTracker signals after the perspective PSC lines were treated with menadione to increase ROS. Scale bar, 20 μm for all.

Since *OPA1* mutations may result in elevation of cellular reactive oxygen species (ROS), we performed live cell imaging to monitor mitochondria and detect ROS in DOA patients’ iPSC lines and a control H9 ESC line. A similar distribution of mitochondria was observed in 1iDOA, 2iDOA, and the control H9 using MitoTracker Red (Figure 4). However, elevated ROS signals were detected in 2iDOA cells compared to 1iDOA or H9 cells (Figures 4A, C, E). When the cells were treated with a ROS elevating reagent menadione, all PSC lines showed increased ROS levels (Figures 4B, D, F). Further detailed analysis will be necessary to determine the mechanism of the differential ROS production and whether mitochondrial function in PSC-derived neurons are affected.

## Discussion

Although OPA1 function has been extensively studied, it remains an enigma why retinal projection neuron RGCs are particularly prone to degenerate when OPA1 function is compromised, given that OPA1 is a ubiquitously expressed mitochondrial protein. One significant roadblock in the past has been the scarcity of human RGCs. Over the last decade, human PSCs have emerged as replenishable sources to produce different somatic tissue and cell types. Since DOA has not been studied in-depth using human RGCs, stem cell-based models provide unprecedented opportunities to investigate DOA disease mechanisms. However, given the molecular and clinical heterogeneity of the DOA patient population and incomplete penetrance of *OPA1* mutations, it is unlikely that an *in vitro* model from PSCs containing one specific *OPA1* mutation will be able to recapitulate the full spectrum of the disease. The iPSC lines from the two DOA patients reported here carry *OPA1* mutations distinct from those in previously reported iPSC lines (Chen et al., 2016; Galera-Monge et al., 2016; Zurita-Diaz et al., 2017; Jonikas et al., 2018; Zhang XH. et al., 2021; Chan et al., 2022; Sladen et al., 2022; Sun et al., 2022), thus providing additional tools to study DOA using PSC-based human RGC models. Furthermore, the clinical records and DOA-associated ophthalmological symptoms for each DOA patient at the time of iPSC derivation will be useful information when comparing *in vitro* cellular level findings with clinical manifestations.

The two DOA patients’ heterozygous *OPA1* mutations do not affect the mitochondrial targeting or alternative splicing of *OPA1*, but are predicted to produce truncated proteins. Ophthalmic examinations showed that Patient 1, whose mutation falls within the central dynamin region of OPA1 (p.Glu650Glyfs*4), has more severe and extensive DOA symptoms. Previous studies have established iPSC lines from DOA+ patients with mutations in the central dynamin region (Galera-Monge et al., 2016; Zurita-Diaz et al., 2017), suggesting a correlation of this regions with a more severe disease pathology. Interestingly, Patient 2 has a frameshift mutation (p.Ile473Phefs*12) that leads to a stop codon within the GTPase domain. Missense mutations in the GTPase domain have been shown to be more likely to cause DOA+ (Hoyt, 1980; Treft et al., 1984; Amati-Bonneau et al., 2008; Yu-Wai-Man et al., 2010b; Maeda-Katahira et al., 2019). However, Patient 2 has remained largely asymptomatic. This suggests that OPA1 proteins with a mutated GTPase domain may act differently from OPA1 proteins with a partially truncated GTPase domain. The mutation carried by 2iDOA iPSC lines is different from the other iPSC lines previously generated containing mutations in the GTPase domain (Sladen et al., 2021; Chan et al., 2022; Sun et al., 2022). To our knowledge, this also is the first report of iPSC line generated from OPA1-DOA patient with a mild symptom. Intriguingly, the initial characterization of 2iDOA PSCs has revealed higher cellular ROS compared to 1iDOA PSCs or the control H9 ESCs. This suggests that under *in vitro* culture conditions, effects of OPA1 mutations may be more readily detected.

OPA1 protein is known to undergo proteolytic processing upon entry into mitochondria and to form protein complexes (Ishihara et al., 2006; Song et al., 2007; Anand et al., 2014; Del Dotto et al., 2017). Despite the autosomal dominant inheritance pattern of DOA, the disease pathogenicity can occur via dominant negative or haploinsufficiency mechanisms, depending on the type and location of the *OPA1* mutations (Delettre et al., 2001). The availability of *OPA1* mutant iPSC lines can benefit the further characterization of *OPA1* mutant iPSC-derived RGCs and shed light on DOA disease mechanisms, especially regarding the suitability of different types of molecular therapies. In the case of haploinsufficiency, gene supplement therapy providing wild type OPA1 is expected to rescue neuronal deficiencies. On the other hand, for dominantly acting *OPA1* mutations, alternative approaches such as RNAi knock-down or CRISPR gene editing can be designed to dampen or eliminate the dominant interfering effects of a given mutant. Therefore, establishing *in vitro* OPA1-DOA disease models is important for developing molecular therapies and enabling personalized medicine.

Increasing evidence indicates that mitochondrial dysfunction plays an important role in neurodegenerative and aging-related diseases (Carelli et al., 2017; Iannielli et al., 2018). Findings from *OPA1*-mutant iPSC-derived disease models can advance the understanding of the pathological mechanisms underlying DOA as well as provide important insights into other neurodegenerative diseases that share common metabolic deficiencies with DOA.

## Methods

### Patient enrollment and ophthalmic examinations

This research was conducted at the University of California, Los Angeles under the institutional review board (IRB) protocol #19-000879, and approved by the Office of the Human Research Protection Program (OHRPP). All participants signed informed consent and Health Insurance Portability and Accountability Act (HIPAA) research authorization forms to enroll in the study. Participants were outpatients of the Doheny Eye Clinics of UCLA. Detailed clinical data including family history, visual acuity, tonometry, fundus photography (Optos), Humphrey visual field (Zeiss), and optical coherence tomography (Patient 1, Heidelberg Engineering; Patient 2, Zeiss) was collected.

### Generation of DOA patient iPSCs

Peripheral blood was collected from DOA patients the same day the ophthalmologic tests were performed. Peripheral blood mononuclear cells (PBMCs) were reprogrammed into iPSCs at the Cedars Sinai Medical Center iPSC core using a non-integration method as previously described (Toombs et al., 2020). Two independent iPSC lines were established for each DOA patient. The iPSC lines were named as 1iDOAn1a and 1iDOAn1b for Patient 1, and 2iDOAn1a and 2iDOAn1b for Patient 2, respectively. The characterization included *mycoplasma* testing, alkaline phosphatase staining, G-band karyotyping, and short-tandem repeat (STR) cell line authentication. Lines are referred to as 1iDOA and 2iDOA throughout this manuscript, but experiments described primarily used n1a clones. 1iDOA carries the heterozygous *OPA1* mutation NC_000003.12:g.193648807dup and 2iDOA carries the heterozygous *OPA1* mutation NC_000003.12:g.193643567_193643568del.

### Human PSC cell cultures

Human ESCs and iPSCs were maintained in mTeSR^TM^ Plus medium (Stemcell Technologies) supplemented with 1% Antibiotic Antimycotic (Gibco/ThermoFisher Scientific) on Matrigel (Corning) coated plates at 37°C with 5% CO_2_. PSCs were passaged by dissociating monolayer cells into a single cell suspension with Accutase (Stemcell Technologies). Single cells were plated in mTeSR^TM^ Plus supplemented with 1% Antibiotic Antimycotic and 10 μM of Y-27632 (Stemcell Technologies) for 24 h, after which the medium was replaced with mTeSR^TM^ Plus with 1% Antibiotic Antimycotic. The medium was changed no less frequently than every other day.

### Immunofluorescent staining and imaging

PSCs grown on Matrigel-coated NUNC™ Thermanox™ 13 mm plastic coverslips (ThermoFisher Scientific) were fixed in 4% paraformaldehyde in PBS for 2 min and then incubated in blocking solution (0.1% TritonX-100, 2% donkey serum, 10% FBS in DMEM). Coverslips were incubated with primary antibodies, followed by secondary antibodies and DAPI diluted in blocking solution (Supplementary Table S1). All incubations were for 1 h at room temperature, and staining periods were followed by three 5-min washes in PBS with 0.1% Tween 20. Coverslips were mounted on glass slides (Fisher Scientific) with mounting media (Fluro-Gel, Electron Microscopy Sciences) and imaged using an Olympus BX61 scanning laser confocal microscope with Plan-APO objectives.

### Sequencing of genomic PCR products

Prior to enrolling in the study, each DOA patient’s specific *OPA1* mutation was classified/diagnosed via exon sequencing. To confirm the presence of these mutations in the DOA patients’ iPSC lines, genomic DNA was isolated from iPSCs using the Purelink genomic DNA Mini Kit (Invitrogen). 200 ng of DNA was used as templates in PRCs using Hot Star Taq DNA Polymerase (Qiagen) and primers flanking the respective *OPA1* mutation site (Supplementary Table S2). Thermocycler conditions were as follows: 15 min at 95°C; 35 cycles of 30 s at 94°C, 30 s at 59°C and 1 min at 72°C; and 10 min at 72°C. PCR products were sequenced by Sanger method using the primers listed in Supplementary Table S2.

### Cellular ROS and mitochondria imaging

PSCs grown as a monolayer were washed once with PBS, followed by incubating in mTeSR^TM^ Plus medium containing 5 μM CellROX Green (Invitrogen), 250 nM MitoTracker Red (Invitrogen), and 5 μg/mL Hoechst 33342 at 37°C for 30 min. A different set of PSC cultures were incubated with 100 μM menadione at 37°C for 1 h prior to detection using CellROX and MitoTracker. After washing with PBS and HBSS without phenol red, cells were immediately imaged using an Olympus IX81 scanning laser confocal microscope with Plan-APO objectives. All images were captured under identical conditions.

## Data Availability

The original contributions presented in the study are included in the article/Supplementary Material, further inquiries can be directed to the corresponding authors.
