# Supplementary material for: Establishing induced pluripotent stem cell lines from two dominant optic atrophy patients with distinct OPA1 mutations and clinical pathologies
Source: Front Genet. 2023 Sep 4;14:1251216. doi: 10.3389/fgene.2023.1251216 (PMC10513078; doi:10.3389/fgene.2023.1251216)
Supplement: Supplementary file 1 [file Table1.pdf]

Supplementary Table 1. Antibodies and Chemical Reagents				
Type	Name	Company	Catalog Number	Dilution or Final Concentration
Primary Antibody	SOX2	Santa Cruz	sc17320	1:50
	NANOG	abcam	ab21624	1:100
	OCT3/4 (C-10)	Santa Cruz	sc5279	1:50
Secondary Antibody	Donkey α goat Alexa 488	Invitrogen	A-11055	1:500
	Donkey α rabbit Alexa 647	Invitrogen	A-31573	1:500
	Donkey α mouse Alexa 594	Invitrogen	A-21203	1:500
Other Reagents	DAPI	Sigma	D9542	10 μg/mL
	Hoechst 33342	Invitrogen	H3570	5 μg/mL
	MitoTracker Red CMXRos	Invitrogen	M7512	250 nM
	CellROX Green Reagent	Invitrogen	C10444	5 μM
	Menadione	Sigma	M5625	100 μM

Supplementary Table 2. PCR and Sequencing Primers		
Exon 19	Forward, Sequencing	5’—CCTCCCTTTGGTTATCTCTG—3’
	Forward, PCR	5’—CACATAACGTGAACAAGTGT—3’
	Reverse, PCR and Sequencing	5’—TACTCAGAATGGAGAACCTG—3’
Exon 12/13	Forward, PCR and Sequencing	5’—GTGAGCGTCTTATCTGAATGG—3’
	Reverse, PCR and Sequencing	5’—CCCCTGTGTCTACATTATAGC—3’
